# Moxifloxacin‐Induced Bullous Acute Generalized Exanthematous Pustulosis (AGEP): A Case Report of a Rare Clinical Presentation

**DOI:** 10.1155/crdm/4921907

**Published:** 2026-09-18

**Authors:** Mehmet Yeral, Dilek Dasgin, Ceyda Tetik Aydogdu, Ekin Altinbas, Serkan Yasar Celik, Baris Bat

**Affiliations:** ^1^ Department of Dermatology, Mugla Sitki Kocman University Faculty of Medicine, Training and Research Hospital, Mugla, Turkey, onkoloji.gov.tr; ^2^ TC Ministry of Health Mugla Training and Research Hospital, Mugla, Turkey; ^3^ Department of Pathology, Mugla Sitki Kocman University Faculty of Medicine, Training and Research Hospital, Mugla, Turkey, onkoloji.gov.tr

**Keywords:** acute generalized exanthematous pustulosis (AGEP), bullous AGEP, drug eruption, moxifloxacin, pustulosis, severe cutaneous adverse reaction (SCAR), toxic epidermal necrolysis (TEN)

## Abstract

Acute generalized exanthematous pustulosis (AGEP) is a severe cutaneous adverse reaction (SCAR) typically characterized by sterile pustules. The bullous variant of AGEP is exceptionally rare and represents a significant diagnostic challenge as it clinically mimics toxic epidermal necrolysis (TEN). We report the case of a 91‐year‐old woman who developed AGEP following moxifloxacin therapy, which rapidly progressed into a bullous form. A striking and rarely described clinical phenomenon—pustules developing directly on the roof of flaccid bullae—was observed. To our knowledge, this is the first reported case of the bullous variant of AGEP specifically triggered by moxifloxacin. Despite intensive treatment, the patient succumbed to sepsis on Day 15. This case underscores the importance of recognizing atypical AGEP presentations to avoid diagnostic confusion with Stevens–Johnson syndrome (SJS)/TEN and highlights a novel severe reaction to a widely used fluoroquinolone.

## 1. Introduction

Acute generalized exanthematous pustulosis (AGEP) is a severe drug‐induced skin eruption characterized by the acute onset of numerous nonfollicular sterile pustules on an erythematous base [1]. Antibiotics are the most common triggers of AGEP, although a wide variety of medications have been implicated [2]. Atypical presentations with vesicles, bullae, targetoid lesions, or purpura may occur, although they are rare [3].

Severe or bullous presentations of AGEP are clinically significant because vesicles, bullae, and extensive skin detachment may closely mimic toxic epidermal necrolysis (TEN), creating substantial diagnostic and therapeutic challenges [3]. While an extensive range of medications has been documented in the literature as potential triggers for AGEP, fluoroquinolones represent one of the recognized drug classes associated with this reaction. In a comprehensive review of 297 patients, 154 unique drugs were identified as suspect triggers, among which fluoroquinolone antibacterials were specifically reported [4]. While certain agents in this class, such as ciprofloxacin, have been reported to trigger AGEP presentations that mimic bullous drug eruptions [5], this specific bullous association has never been described for moxifloxacin. Although moxifloxacin is a recognized etiologic agent for conventional pustular AGEP [6], the bullous variant has not been previously documented in association with moxifloxacin therapy. Consequently, our findings represent the first reported case of a primary bullous phenotype specifically induced by moxifloxacin to date.

We present a diagnostically challenging and visually striking case of bullous AGEP featuring the rarely described “pustules‐on‐bullae” sign.

## 2. Case Presentation

A 91‐year‐old female presented to the emergency department with fever, nausea, and vomiting. Laboratory investigations revealed a significant inflammatory response (CRP: 128 mg/L, WBC: 59,940/μL with 95% neutrophils) and acute kidney injury (creatinine: 1.24 mg/dL). Urinalysis confirmed a urinary tract infection. There was no personal or family history of psoriasis, psoriatic arthritis, or other pustular skin disorders, and the patient had no prior history of scaly erythematous plaques, nail dystrophy, or previously documented drug eruption. The patient was admitted to the internal medicine ward, and intravenous moxifloxacin was initiated.

**FIGURE 1:**
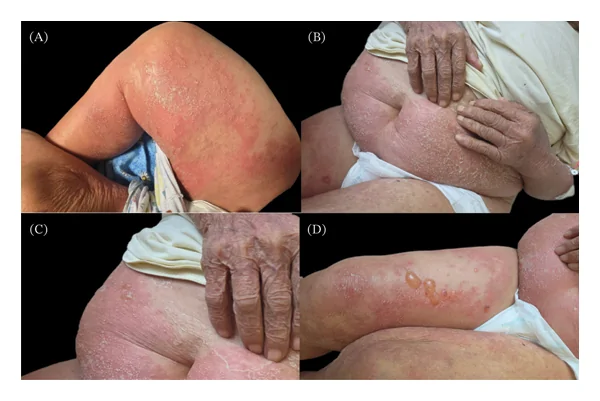
Clinical presentation of AGEP and its bullous evolution. (A) Widespread pustular eruption on an erythematous base, consistent with AGEP. (B) Confluent pustular rash on the abdomen. (C) Emergence of bullae on the flank. (D) Multiple bullae on the lower extremities, in addition to the existing erythematous pustules.

On Day 2 of moxifloxacin therapy, the patient developed a widespread, burning, erythematous rash covered with hundreds of small pustules on the trunk (Figure 1). A dermatology consultation was requested, and a preliminary diagnosis of AGEP was made. Moxifloxacin was immediately discontinued, and systemic corticosteroid therapy at 0.5 mg/kg/day was started. A skin biopsy was performed. The initial biopsy result was consistent with AGEP.

**FIGURE 2:**
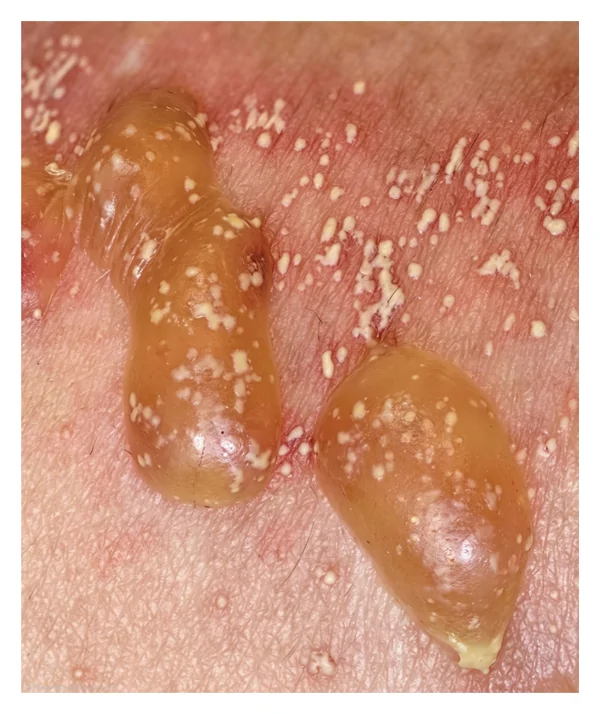
A close‐up view demonstrating the characteristic finding of new pustules developing directly on the roof of a flaccid bulla.

On Day 5, the clinical picture evolved dramatically. New, flaccid bullae appeared over the preexisting erythematous areas. Most strikingly, numerous sterile pustules were observed developing on the roofs of these bullae, creating a rarely described “pustules‐on‐bullae” appearance (Figure 2). Given the rapid progression and skin detachment, the differential diagnosis expanded to include TEN, Stevens–Johnson syndrome (SJS)/TEN overlap, and generalized bullous fixed drug eruption (GBFDE).

**FIGURE 3:**
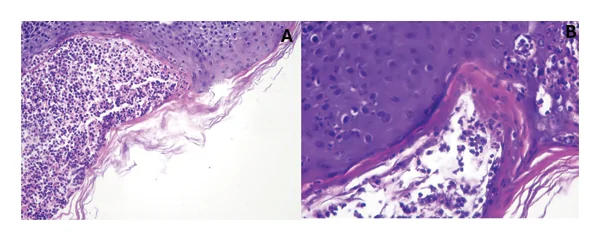
Histopathological findings (hematoxylin & eosin, 200x). (A) A subcorneal neutrophilic microabscess in the epidermis. (B) An intraepidermal neutrophilic pustule (top right).

A second set of biopsies for histopathology and direct immunofluorescence (DIF) was obtained. Histopathology demonstrated classic AGEP features: subcorneal and intraepidermal neutrophilic pustules, spongiosis, and a perivascular inflammatory infiltrate with neutrophils and eosinophils, without the full‐thickness epidermal necrosis seen in TEN (Figure 3). DIF was negative, ruling out bullous pemphigoid. Despite dermatological improvement with steroid therapy, the patient’s systemic condition worsened due to advanced age and comorbidities; she passed away on Day 15 due to sepsis.

## 3. Discussion

Bullous AGEP is an exceptionally rare phenotype that represents an atypical variant within the clinical spectrum of AGEP. The distinguishing feature of our case is the striking clinical manifestation of pustules overlying bullae. Given that bullous AGEP is exceedingly rare in the literature, the exact pathophysiology of bulla formation is not yet fully elucidated. However, we hypothesize that this presentation reflects a dual pathological process: the formation of characteristic intraepidermal neutrophilic pustules alongside intense papillary dermal edema. This profound edema likely results in mechanical subepidermal separation at the dermoepidermal junction, subsequently leading to the clinical development of bullae [5, 7].

An important differential diagnosis of AGEP is generalized pustular psoriasis (GPP), particularly given the sudden onset of numerous nonfollicular pustules on an erythematous base. However, several features argued against GPP in our case: the patient had no personal or family history of psoriasis or psoriatic arthritis; there was no history of preceding scaly, erythematous plaques or nail dystrophy; the eruption occurred with a clear temporal association to moxifloxacin initiation (within 48 h of drug exposure); and histopathology demonstrated the characteristic features of AGEP without the psoriasiform hyperplasia or Munro microabscesses typically seen in psoriasis [2]. Moreover, the clinical morphology of the pustular eruption is also distinct from that of pustular psoriasis; in addition, the presence of flaccid bullae would not be expected in pustular psoriasis, further supporting our diagnosis. The rapid clinical clearance following drug withdrawal, together with the histopathological findings and negative DIF, further supported a diagnosis of drug‐induced AGEP rather than acute GPP.

Distinguishing bullous AGEP from TEN is critical due to different prognostic and management implications. While TEN typically involves mucosal surfaces and shows full‐thickness epidermal necrosis, bullous AGEP is characterized by subcorneal pustules and a more favorable cutaneous prognosis if the trigger is removed [8]. In our case, the “pustules‐on‐bullae” sign served as a strong clinical clue for AGEP, subsequently confirmed by histopathology. Notably, bullous impetigo may also present with pustules and bullae; however, the absence of clinical features of infection at the site, the widespread symmetric distribution, the sterile nature of the pustules, and the histopathological pattern effectively excluded this differential.

Fluoroquinolones are known but are infrequent triggers for AGEP. While Häusermann et al. described a ciprofloxacin‐induced case mimicking bullous eruption [5], moxifloxacin has been primarily linked to classic AGEP or GBFDE [6, 9]. Although primary bullous variants are exceedingly rare and have been documented with other agents such as terbinafine [10], our report stands as the first documented case of a moxifloxacin‐induced bullous variant of AGEP.

To ensure diagnostic accuracy, the Euro severe cutaneous adverse reaction (SCAR) (EuroSCAR) AGEP validation score was calculated for our patient. The EuroSCAR criteria, developed by Sidoroff et al. [1], comprise a structured scoring algorithm that integrates morphological features (pustules, erythema, distribution, and postpustular desquamation), clinical course (mucosal involvement, acute onset within 10 days, resolution within 15 days, fever ≥ 38°C, and peripheral neutrophilia > 7000/μL), and histopathological findings. The total score is interpreted as follows: ≤ 0, no AGEP; 1–4, possible; 5–7, probable; and 8–12, definite AGEP. In our patient, the score was calculated as follows: typical pustular morphology (+2), typical erythema (+2), typical generalized distribution (+2), absence of mucosal involvement (0), acute onset within 48 h of drug exposure (0), fever ≥ 38°C (+1), marked peripheral neutrophilia (WBC: 59,940/μL with 95% neutrophils; +1), and histopathology showing spongiform subcorneal and intraepidermal pustules with papillary dermal edema (+3). This yielded a total score of 11 out of a maximum of 12, consistent with a definite diagnosis of AGEP. The score sheet items pertaining to postpustular desquamation and resolution within 15 days were conservatively scored as 0 due to the patient’s death from sepsis on Day 15, which precluded full documentation of these outcomes despite dermatological improvement being observed prior to death.

Although AGEP generally carries a favorable prognosis, with the eruption typically resolving spontaneously upon withdrawal of the offending agent [2], mortality has been reported. In the multinational EuroSCAR case–control study of 97 validated AGEP cases, Sidoroff et al. reported a mortality rate of approximately 4%, occurring almost exclusively in elderly patients with significant underlying comorbidities and secondary infections [11]. Overall mortality in AGEP is generally considered to be below 5%, with fatal outcomes attributed mainly to multiple organ dysfunction and occurring particularly in patients with significant comorbidities or extensive disease [2, 11]. A significant proportion of these fatal cases involve elderly individuals with critical preexisting conditions [4]. Our patient exemplified this reported high‐risk profile: she was 91 years old, presented with acute kidney injury and a coexisting urosepsis at the time of admission, and ultimately succumbed to sepsis on Day 15. Importantly, her dermatological condition was improving prior to death, suggesting that the fatal outcome primarily reflected extracutaneous factors (advanced age, comorbidities, and concurrent urosepsis) rather than the bullous phenotype itself.

In summary, we report a rare and visually striking case of moxifloxacin‐induced bullous AGEP. The “pustules‐on‐bullae” sign observed in this patient represents a rarely described clinical finding that may aid clinicians in distinguishing bullous AGEP from TEN. To our knowledge, this is the first reported case in the literature to identify moxifloxacin as the causative agent for this bullous variant of AGEP, and the EuroSCAR score of 11 confirms a definite diagnosis. While conventional AGEP is often characterized by a self‐limiting course and a relatively low mortality rate of less than 5% [2, 11], the fatal outcome in our patient is best explained by the interplay of advanced age, multiple comorbidities, and concurrent urosepsis, rather than the bullous phenotype per se. Given that the number of reported bullous AGEP cases remains too small to draw firm conclusions regarding prognostic implications, further observations are needed to determine whether the bullous phenotype carries a distinct mortality risk. Nevertheless, clinicians should maintain a high level of vigilance and ensure close monitoring when prescribing moxifloxacin to elderly patients with significant comorbidities.

## Author Contributions

Mehmet Yeral: conceptualization, data curation, writing–original draft, and patient care.

Dilek Dasgin: supervision, data curation, patient care, and writing–review and editing.

Ceyda Tetik Aydogdu: data curation, patient care, and writing–review and editing.

Ekin Altinbas: data curation, patient care, and writing–review and editing.

Serkan Yasar Celik: histopathological analysis, data curation, and writing–review and editing.

Baris Bat: histopathological analysis, supervision, and writing–review and editing.

## Funding

No funding was received for this study.

## Ethics Statement

The study protocol was reviewed, and exemption was granted by the institutional review board of Mugla Sitki Kocman University. The study was conducted in accordance with the Declaration of Helsinki.

## Consent

Written informed consent was obtained from the patient’s next of kin (family) for the publication of this case report and any accompanying images.

## Conflicts of Interest

The authors declare no conflicts of interest.

## Data Availability

The data that support the findings of this study are available on request from the corresponding author. The data are not publicly available due to privacy or ethical restrictions.
